# Acknowledgement to Referees

**DOI:** 10.1186/s40798-015-0039-3

**Published:** 2015-12-29

**Authors:** Steve McMillan, Roger Olney

**Affiliations:** Springer Science + Business Media, 5 The Warehouse Way, Auckland, Northcote New Zealand

Dear Reader,

As we approach the end of 2015 and the completion of the first ever volume of *Sports Medicine - Open*, the editors and production staff at SpringerOpen wish to reflect on a successful year’s achievements and pay thanks to all those who have contributed their time and effort to establish this new journal.

We have been delighted by the way the sports science community has embraced the new journal and grateful for the number of high-quality submissions that the journal has received. The inaugural volume of *Sports Medicine - Open* will close with more than 20 original research articles, a dozen review articles (including systematic reviews), one particularly well-downloaded current opinion piece, and two editorials. And while we must offer our sincere thanks to all the authors who have contributed thus far, the quality of published articles is, similarly, testament to the significant efforts of the peer reviewers, whose commitment ensures that our content is held to the highest possible standard.

In addition to the members of our Honorary Editorial Board, we would like to thank the following individuals who have acted as reviewers for *Sports Medicine - Open*, since the journal began receiving submissions in mid-2014:

Roger Adams, Australia

Maicon R. Albuquerque, Brazil

Roy D. Altman, USA

Isabel Andia, Spain

Sophie Attwood, UK

Filippo Aucella, Italy

Astrid Balemans, the Netherlands

Kristoffer Barfod, Denmark

Michelle Barrack, USA

Parveen Bawa, Canada

Daniel A. Boullosa, Brazil

Christian Brinkmann, Germany

Diego Brunelli, Brazil

Robert Buresh, USA

Robin Callister, Australia

Helmi Chaabène, Tunisia

Karim Chamari, Qatar

Jean-Claude Chatard, France

Kong Y. Chen, USA

Hsiu-Ching Chiu, Taiwan

Demetra D. Christou, USA

Hamdi Chtourou, Tunisia

Imogen Nicola Clark, Australia

Mike Climstein, Australia

Brian J. Cole, USA

Mark Connick, Australia

Ricardo Costa, Australia

Antonio Crisafulli, Italy

Nicole C. Dabbs, USA

Abdolhamid Daneshjoo, Iran

J. Derek Kingsley, USA

Beat Knechtle, Switzerland

Karsten Koehler, USA

Jan Konarski, Poland

Nikolaos E. Koyndoyrakis, Greece

Michael Lamont, New Zealand

Emilio Landolfi, Canada

Robert Lansing, USA

Steen Larsen, Denmark

David Lavallee, UK

Heather Leach, Canada

Jill Leckey, Australia

Ross K. Leighton, Canada

Shannon Lennon-Edwards, USA

Xinning Li, USA

Harry Lim, Singapore

Adriano E. Lima da Silva, Brazil

Alexis Lion, Luxembourg

Grant S. Lipman, USA

Fabio Lira, Brazil

Jeremy P. Loenneke, USA

Alfredo M. Lurati, Italy

Theresa Mann, South Africa

Frank Marino, Australia

Myosotis Massidda, Italy

James P. McClung, USA

Jane McDevitt, USA

Mike McGuigan, New Zealand

Antti Mero, Finland

Kevin Miller, USA

Chris Mills, UK

Bartosz Molik, Poland

Nicholas Murray, USA

Kathy Myburgh, South Africa

Ara Nazarian, USA

Mathieu Nédélec, France

Jeanne F. Nichols, USA

Sean Davies, USA

Philip Davis, UK

Benedito S. Denadai, Brazil

Stefano D'Ottavio, Italy

Wong Jyh Eiin, Malaysia

Jennifer Etnier, USA

Nir Eynon, Australia

Irene Faber, the Netherlands

Éanna Falvey, Ireland

Rômulo Fernandes, Brazil

Jared Fletcher, Canada

Maria Francesca, Qatar

Tim Gabbett, Australia

Jarred Gillett, Australia

Paul Glazier, UK

Urs Granacher, Germany

Tyson Grier, USA

Jordan Guenette, Canada

Esther Hartman, the Netherlands

Keith G. Hauret, USA

Jaime Hinzpeter, Chile

Martin D. Hoffman, USA

Chun-Jung Huang, USA

Jasmin Hutchinson, USA

Xanne Janssen, UK

Yong-Seok Jee, Republic of Korea

Uffe Jørgensen, Denmark

Jeremy D. Joslin, USA

Liz Joy, USA

Jyrki Kettunen, Finland

David Nieman, USA

Onni Niemelä, Finland

Vincent Nougier, France

Michał Nowicki, Poland

Ari Nummela, Finland

Michael P. Nyberg, Denmark

Rob M. Orr, Australia

Debbie Palmer-Green, UK

Andy Pasternak, USA

Charles Pedlar, UK

Jesper Petersen, Denmark

Maria Francesca Piacentini, Italy

Paul Pillitteri, USA

Flávio Pires, Brazil

Claudia L. Reardon, USA

E. Justy Reed, USA

Chris Rhea, USA

William O. Roberts, USA

Alejandro Santos-Lozano, Spain

Dean K. Simonton, USA

Sabrina Skorski, Germany

Andrew Springer, USA

Cathy Starr, USA

Laura Stewart, USA

Thomas Swensen, USA

John Temesi, Canada

Masaru Teramoto, USA

Robert Thiebaud, USA

Kevin Till, UK

Argyris Toubekis, Greece

Tinna Traustadóttir, USA

Edith Van Dyck, Belgium

Geert Verheyden, Belgium

Megan Wenner, USA

Nancy Williams, USA

Jinliang Xing, China

James Zois, Australia

We hope that you have found the articles published this year to be both interesting and informative, and we look forward to keeping you up to date with more sport and exercise science research in 2016.

With best wishes from the staff of *Sports Medicine - Open* and everyone at SpringerOpen.

